# *Casearia napoensis* (Salicaceae), a new species from Guangxi, China

**DOI:** 10.3897/phytokeys.279.206429

**Published:** 2026-09-30

**Authors:** Ting Xu, Zilong Ouyang, Zhiyong Su, Mei Yang, Xianglu Jia, Li Gong

**Affiliations:** 1 Nanning Botanical Garden, Nanning 530002, China Nanning Botanical Garden Nanning China; 2 Nanning Qingxiu Mountain Scenic Area Tourism Development Co., Ltd., Nanning 530004, China Nanning Qingxiu Mountain Scenic Area Tourism Development Co., Ltd. Nanning China; 3 Nanning Horticultural Expo Garden Administration Center, Nanning 530021, China Nanning Horticultural Expo Garden Administration Center Nanning China

**Keywords:** *
Casearia
*, Guangxi, morphology, new species, Salicaceae

## Abstract

*Casearia napoensis***sp. nov**. a new species from Guangxi, Southern China, is described and illustrated. The new species exhibits several distinguishing features: capsule 2.2–3 cm, spherical, leathery, orange-red at maturity, 4- or 5-valved, valves fleshy, 0.5 cm thick. Seeds up to 15–16 per fruit. The relationships among *C. napoensis*, *C. membranacea* and *C. tardieuae* are shortly discussed. The new species is endemic to limestone mountain forests of Western Guangxi, with its known distribution largely confined to the Guangxi Laohutiao Nature Reserve. On the basis of the IUCN criteria, it is assessed as Critically Endangered.

## Introduction

*Casearia* Jacq. is a pantropical woody genus comprising approximately 200 species, with its greatest diversity occurring in the Neotropics (de Mestier et al. 2023). It is generally placed in Salicaceae (Chase et al. 2002; APG IV 2016), though Alford (2005) proposed a plausible classification that would segregate *Casearia* and about a dozen other genera of Salicaceae into Samydaceae (Philpott and Applequist 2023). *Casearia* comprises seven species in China, these seven species are characterized by: flowers arranged in axillary glomerules; disk lobes in same row as stamens; style entire; capsule fleshy (Flora of China Editorial Committee 2007).

Recent discoveries of new *Casearia* species have been largely concentrated in Southeast Africa (Philpott and Applequist 2023), Central America (de Mestier et al. 2023), South America (Marquete and Torres 2022) and West Africa (Breteler and Baldé 2024). Since the publication of the Flora of China, the taxonomy of *Casearia* in China has remained static, with no new species having been published in the past 20 years. In this paper, we describe a new species of *Casearia* endemic to Southern China, *Casearia napoensis* T. Xu, L. Gong, Z. L. Ouyang & Z. Y. Su.

## Materials and methods

From 2024 to 2026, the research team conducted a total of 13 field surveys at the type locality. Morphological observations and measurements of the new species were carried out on both living plants in the field and herbarium specimens. Comparisons among the *Casearia* species distributed in China were made using relevant literature and digital images of specimens housed at institutions such as AU, BJFC, CSFI, FJSI, GXMG, HITBC, HUH, IBK, IBSC, IMDY, KUN, NAS, P, PE, SWFC, SZG and WUK. In addition, type specimen images were consulted via JSTOR Global Plants. [https://plants.jstor.org] and Chinese Virtual Herbarium Website [http://www.cvh.ac.cn/]. The voucher specimens are deposited in the Herbarium of Nanning Botanical Garden (**NNBG**), the Herbarium of Chenshan Botanical Garden (**CSH**) and the Herbarium of Guangxi Institute of Chinese Medicine & Pharmaceutical Sciences (**GXMI**). The Nanning Botanical Garden collected seeds and successfully raised seedlings from them for ex-situ conservation.

## Taxonomic treatment

### Casearia napoensis

Taxon classification

Plantae

MalpighialesSalicaceae

T.Xu, L.Gong, Z.L.Ouyang & Z.Y.Su sp. nov.

94396E47-1B4E-5FCE-ADC4-8AB18324BFCF

urn:lsid:ipni.org:names:77399863-1

Figs 1, 2

#### Type.

China • Guangxi Zhuang Autonomous Region: Baise City, Napo County, Pingmeng Town, Nianjing Village (Guangxi Laohutiao Nature Reserve), 22°58.97'N, 105°50.60'E, 1200 m, a.s.l., 30 August 2024, *XT et al. 20240830-01*, • the same location, 12 May 2026, *XT et al. 260512-07* (holotype, NNBG!; isotype, CSH!, GXMI!, NNBG!).

**Figure 1.**
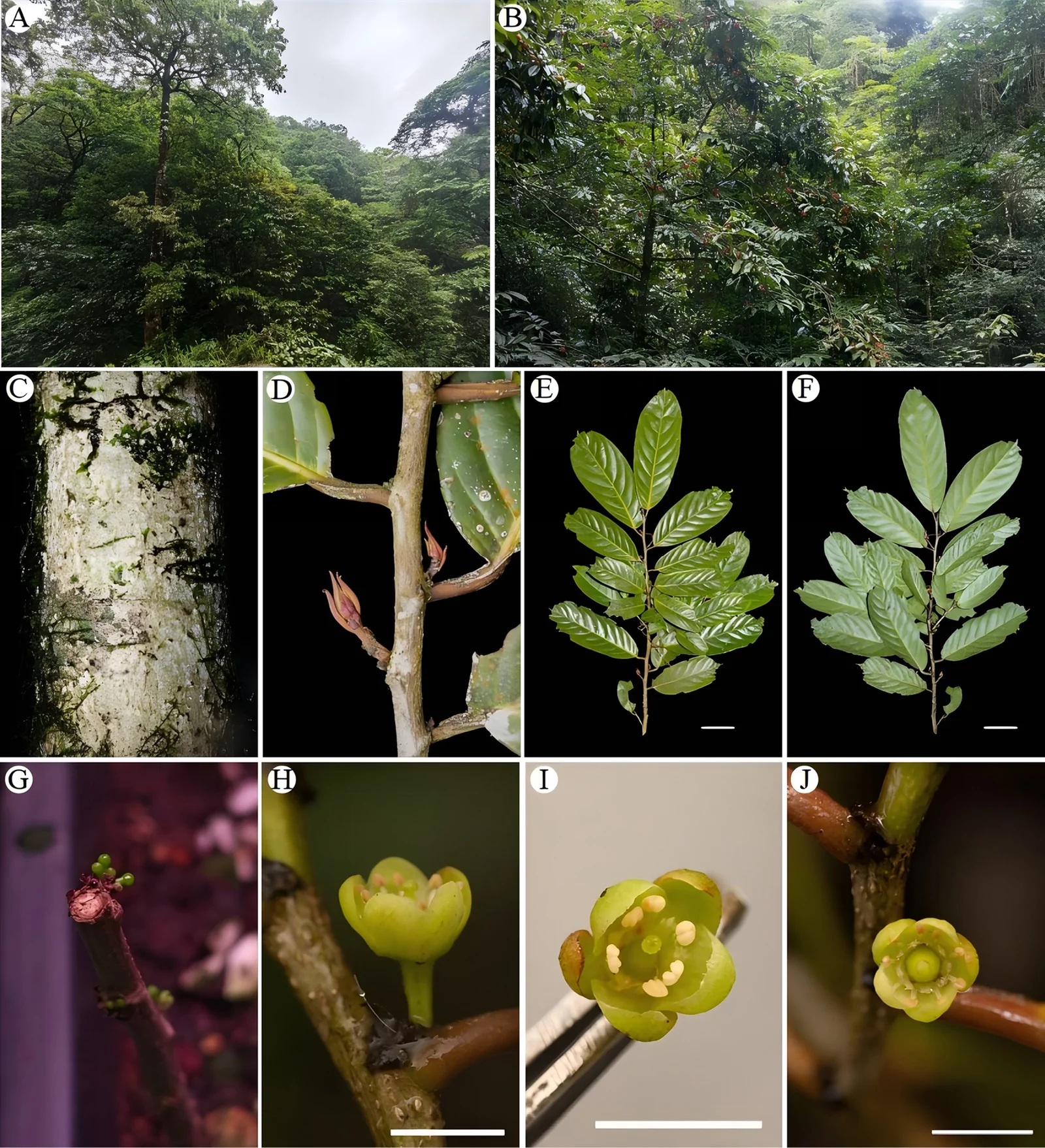
*Casearia napoensis*. **A**. Habitat; **B**. Fruiting plant in habitat; **C**. Bark; **D**. Twig and stipules; **E**. Front side of the leaf; **F**. Back side of the leaf; **G**. Inflorescence; **H**. Flower, views from side; **I**, **J**. Flower, front view. Photo (**G**) by L. Gong; Photos (**A–F**, **H–J**) by T. Xu, photo correction and design by T. Xu. Scale bars: 5.0 cm (**E, F**); 5.0 mm (**H, I, J**).

**Figure 2.**
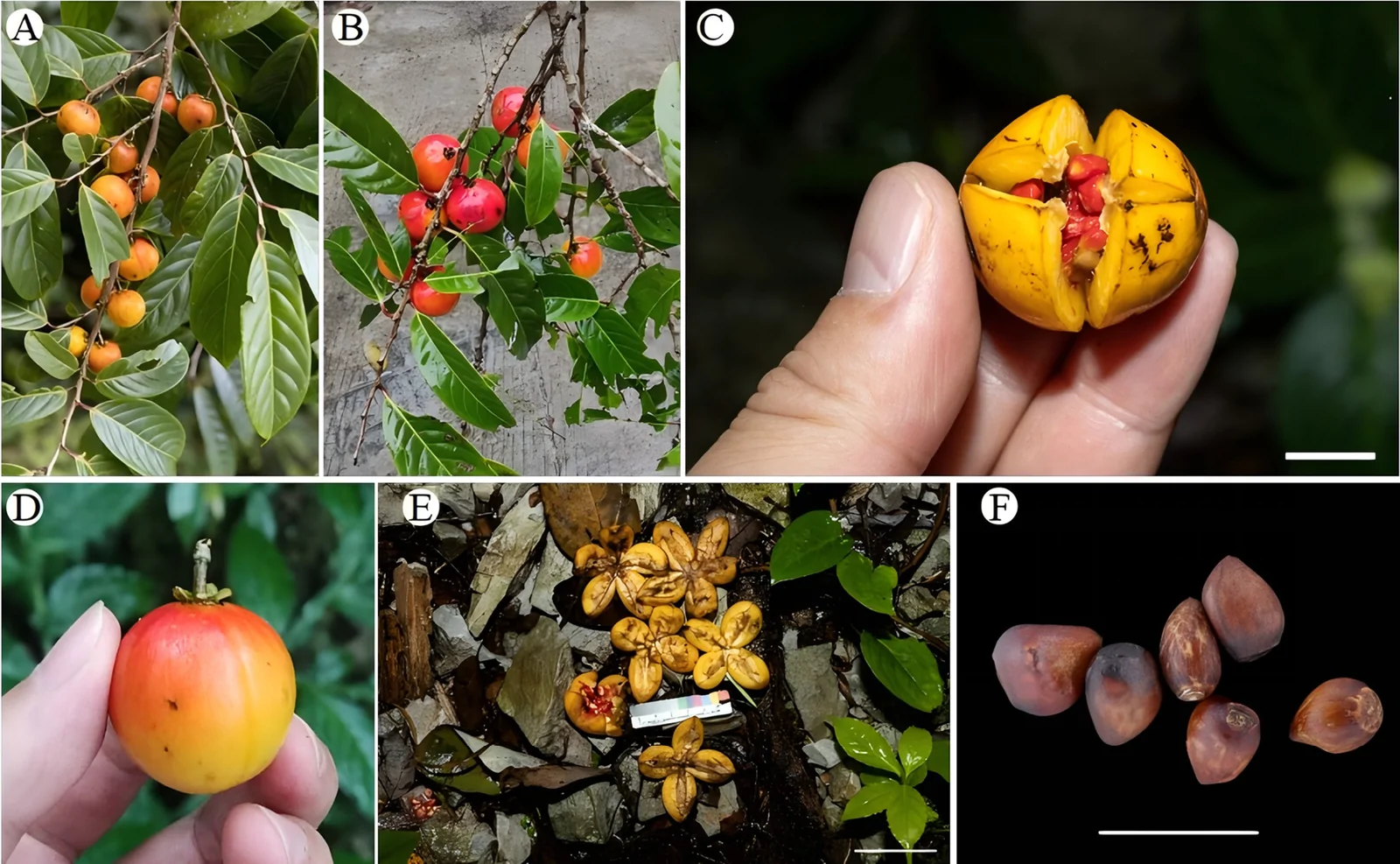
*Casearia napoensis*. **A, B**. Fruiting branch; **C, D**. Ripe fruit; **E**. Dehiscent capsule; **F**. Seeds. Photos (**A**, **C**, **F**) by L. Gong; Photos (**B**, **D**) by Z.L.Ouyang; Photo **E** by T. Xu, photo correction and design by T. Xu. Scale bars: 5.0 cm (**E**); 1.0 cm (**C, F**).

#### Diagnosis.

Similar to *C. membranacea* and *C. tardieuae*, but differs mainly in the stamens 6–9, ca. 2–3 mm, filaments sparsely pubescent. Capsule 2.2–3 cm, spherical, leathery, orange-red at maturity, 4- or 5-valved, valves fleshy, 0.5 cm thick. Seeds up to 15–16 per fruit.

#### Vernacular name.

Chinese: 那坡脚骨脆 (nà pō jiăo gŬ cuì).

#### Description.

Trees or shrubs, 4–8 m tall; trunk straight; bark grayish white. ***Young branches*** longitudinally ridged, exfoliating in flakes, grayish-white, glabrous. ***Stipules*** narrowly triangular, ca. 3 mm, entire, caducous; leaves alternate, thinly leathery, obovate-oblong, 11–15(–16) × 3.5–5.6(–6) cm, apex acuminate, acumen ca. 1 cm, base cuneate, margin entire, occasionally with sparse small glandular teeth on the upper portion, adaxially bright green, abaxially pale green, drying blackish-brown, both surfaces glabrous; lateral veins 8–10 pairs, arching upward, prominent abaxially; petiole 0.7–1.5 cm, glabrous. ***Flowers*** small, bisexual, green, 3–4 clustered in leaf axils, without peduncles, with scale-like bracts at the base; flower diameter ca. 4 mm; ***pedicels*** slender, ca. 3 mm long, glabrous; ***sepals*** 5, ovate, ca. 4 mm long, 3 mm wide, margin membranous and ciliate; ***petals*** absent; stamens 6–9; ***filaments*** sparsely pubescent, ca. 2–3 mm; anthers oblong-ellipsoid; disk lobes oblong, flattened, densely pubescent. ***Capsule*** 2.2–3 cm, spherical, leathery, surface smooth, orange-red when ripe, 4- or 5-valved, valves fleshy, 0.5 cm thick, dried fruit severely shrunken, pericarp thin, dark brown, with 5 longitudinal ridges; ***seeds*** 15–16, white, pale brown when dry, droplet-shaped, 5–7 mm. Fl. Apr–Jun, fr. Aug–Nov.

#### Etymology.

The specific epithet is derived from the county name of the type locality (Napo).

#### Distribution and habitat.

*Casearia napoensis* is only known from the type location, Guangxi, Southern China. Growing in the limestone mountainous area at an altitude of 1200 meters.

#### Conservation status.

*Casearia napoensis* is endemic to the Guangxi Laohutiao Nature Reserve, with a restricted distribution. From May 2024 to May 2026, we conducted 13 field surveys in this area but did not discover any additional localities for the species, suggesting that its distribution is extremely limited. At present, seventeen individuals of *C. napoensis* have been found, including two mature ones. According to the Guidelines for Using the IUCN Red List Categories and Criteria (IUCN Standards and Petitions Committee 2024), *C. napoensis* should be assessed as Critically Endangered (CR) based on criteria D1.

## Discussion

*Casearia napoensis* is distinguished by capsule 2.2–3 cm, spherical, leathery, orange-red at maturity, 4- or 5-valved. Among the recorded species of *Casearia*, this new species is similar to *C. membranacea* and *C. tardieuae*. But both species have 3-valved capsules, which distinguishes them from the new species. The main differences among the three species are summarized in Table 1. In the protologue of *C. tardieuae*, the description states: Ovarium conicum, 2 mm altum. Fructus carnosus ellipsoidalis, 3.5 × 2.5 mm, plus minusve verrucosus (Lescot and Sleumer 1970). This may be a typographical error; the fruit size should probably be 3.5 × 2.5 cm. The two species differ in whether the pericarp is verrucosus, allowing easy distinction from *C. napoensis*. Bao (1983) published *C. calciphila* as a new species. This taxon was later synonymized under *C. tardieuae* in Flora of China (Flora of China Editorial Committee 2007); however, material of *C. tardieuae* was not seen by the authors of that flora. According to the Editorial Committee of Flora Reipublicae Popularis Sinicae (1999), the capsule of *C. calciphila* is described as ellipsoid, ca. 1.2 cm long, which also serves to differentiate it from *C. napoensis*.

**Table 1.** Morphological comparisons among *Casearia napoensis*, *C. membranacea* and *C. tardieuae*.

**Characters**	***C. napoensis***	***C. membranacea***	***C. tardieuae***
Branches	Young branches ridged, grayish-white, exfoliating in flakes,	Young branches ridged, green	Young branches wrinkled, brownish-gray, older ones flaky
Stipules	Narrowly triangular, ca. 3 mm	Ovate, ca. 1 mm	Triangular, ca. 2 mm
Leaf blade	Thinly leathery	Papery, sometimes thinly so, or slightly leathery	Leathery to thickly leathery
Lateral veins	8–10 pairs	5–7(–10) pairs	6–8 pairs
Sepals	5, ovate, 3 mm, glabrous	5, broadly elliptic, obovate, or obovate-oblong, ca. 2.5 mm, outside rarely sparsely puberulous	5, ovate, 3.5–4.5 mm, glabrous except for sparsely ciliate margin
Stamens	6–9; ca. 2–3 mm; filaments sparsely pubescent	8–10; 1–2 mm; filaments usually glabrous, rarely puberulous	8; ca. 2 mm; filaments sparsely pubescent, glabrescent or glabrous
Disk	Lobes oblong, flattened, densely pubescent	Lobes oblong or triangular, adaxially glabrous, apex densely hairy, hairs white or reddish, long	Lobes oblong, hairy at apex
Capsule	2.5–3 cm, spherical, leathery, pericarp smooth, 4- or 5-valved	1–1.5(–4) cm, ellipsoid to oblong, leathery, 3-valved	3.5 × 2.5 mm (may be a typographical error, correct: 3.5 × 2.5 cm), ellipsoid, fleshy, verrucose, 3-valved
Seeds	15–16, 5–7 mm, red aril.	Several, 4–5 mm, pale yellowish aril.	Not documented

### Key to the *Casearia* species of China

**Table:** 

1	Stipules narrowly lanceolate or linear-lanceolate, 1.5–10 mm	**2**
–	Stipules broadly triangular or broadly ovate, minute, 1–2 mm	**3**
2	Stipules narrowly lanceolate, 5–10 mm, early caducous, on young growth leaving a large conspicuous pale brown scar; leaves with 10–14 pairs of lateral veins; in dried material pellucid dots and streaks throughout leaf blade usually clearly visible at × 10	***C. graveolens***
–	Stipules linear-lanceolate, 1.5–3 mm, persistent for some time; leaves with 5–8 pairs of lateral veins; in dried material pellucid dots and streaks throughout leaf blade not clearly visible at × 10 mag. without holding leaf up to light, nor reddish brown	***C. flexuosa***
3	Abaxial surfaces of mature leaves pubescent, at least along midvein and lateral veins	**4**
–	Abaxial surfaces of mature leaves glabrous	**5**
4	Pedicels 2–4 mm in flower, 5–6 mm in fruit	***C. velutina***
–	Pedicels 5–8 mm in flower, ca. 10 mm in fruit	***C. kurzii***
5	Leaves leathery	**6**
–	Leaves papery to membranous	**7**
6	Capsule 2- or 3-valved	***C. tardieuae***
–	Capsule 4- or 5-valved	***C. napoensis* sp. nov**.
7	Terminal bud, pedicel, and calyx hairy, stamen filaments usually hairy (rarely nearly glabrous); capsule slightly to not at all ridged, pericarp veined, vesicled, vesicles black and shiny in cross-section	***C. glomerata***
–	Terminal bud hairy or glabrous, pedicel, calyx, and stamen filaments usually glabrous (rarely puberulous); capsule usually strongly ridged, pericarp veined but not conspicuously vesicled	***C. membranacea***

## Supplementary Material

XML Treatment for Casearia napoensis

## Additional information

### Artificial Intelligence (AI) use

The authors accept full responsibility for the content of the manuscript, including the disclosure of any use of AI.

No AI tools were used in the preparation of this manuscript.

### Author ORCIDs

T. Xu https://orcid.org/0000-0001-9598-0387

Z. Ouyang https://orcid.org/0009-0005-0787-2615

Z. Su https://orcid.org/0009-0001-3180-4496

M. Yang https://orcid.org/0000-0002-6821-166X

X. Jia https://orcid.org/0009-0006-9143-8885

L. Gong https://orcid.org/0009-0009-3886-561X

### Data availability

All of the data that support the findings of this study are available in the main text.
